# Biocontrol of Two Bacterial Inoculant Strains and Their Effects on the Rhizosphere Microbial Community of Field-Grown Wheat

**DOI:** 10.1155/2021/8835275

**Published:** 2021-01-08

**Authors:** Xiaohui Wang, Chao Ji, Xin Song, Zhaoyang Liu, Yue Liu, Huying Li, Qixiong Gao, Chaohui Li, Rui Zheng, Xihong Han, Xunli Liu

**Affiliations:** ^1^College of Forestry, Shandong Agricultural Universities, No. 61, Daizong Street, Taian, Shandong 271018, China; ^2^Ministry of Agriculture Key Laboratory of Seaweed Fertilizers, Qingdao 266400, China

## Abstract

Biocontrol by inoculation with beneficial microbes is a proven strategy for reducing the negative effect of soil-borne pathogens. We evaluated the effects of microbial inoculants BIO-1 and BIO-2 in reducing soil-borne wheat diseases and in influencing wheat rhizosphere microbial community composition in a plot test. The experimental design consisted of three treatments: (1) *Fusarium graminearum* F0609 (CK), (2) *F. graminearum* + BIO-1 (T1), and (3) *F. graminearum* F0609 + BIO-2 (T2). The results of the wheat disease investigation showed that the relative efficacies of BIO-1 and BIO-2 were up to 82.5% and 83.9%, respectively. Illumina MiSeq sequencing revealed that bacterial abundance and diversity were significantly higher (*P* < 0.05) in the treatment groups (T1 and T2) than in the control, with significantly decreased fungal diversity in the T2 group. Principal coordinates and hierarchical clustering analyses revealed that the bacterial and fungal communities were distinctly separated between the treatment and control groups. Bacterial community composition analysis demonstrated that beneficial microbes, such as *Sphingomonas*, *Bacillus*, *Nocardioides*, *Rhizobium*, *Streptomyces*, *Pseudomonas*, and *Microbacterium*, were more abundant in the treatment groups than in the control group. Fungal community composition analysis revealed that the relative abundance of the phytopathogenic fungi *Fusarium* and *Gibberella* decreased and that the well-known beneficial fungi *Chaetomium*, *Penicillium*, and *Humicola* were more abundant in the treatment groups than in the control group. Overall, these results confirm that beneficial microbes accumulate more easily in the wheat rhizosphere following application of BIO-1 and BIO-2 and that the relative abundance of phytopathogenic fungi decreased compared with that in the control group.

## 1. Introduction

Soil microorganisms are an important factor maintaining the sustainability of agricultural production systems, with the rhizosphere being a critical region supporting the exchange of nutrients between plants and the surrounding soil environment [1, 2]. Microbes accumulate in the rhizosphere and utilize root exudates released by their plant host(s) [3]. In return, plant-associated bacteria can promote plant growth via various mechanisms including (1) supplying nutrients to plants via nitrogen fixation and solubilization of mineral phosphate and potassium [4, 5], (2) competing with pathogens for nutrients and niches [6], (3) suppressing pathogen proliferation by producing secondary metabolites such as antibiotics and hydrolytic enzymes [7], and (4) inducing systemic resistance [8]. Additionally, the generation of 1-aminocyclopropane-1-carboxylate deaminase (ACCD), indole acetic acid (IAA), and siderophores by plant growth-promoting rhizobacteria (PGPR) can directly or indirectly stimulate seedling growth [9, 10]. Contrastingly to beneficial microbes, negative plant–microbial interactions cause yield losses, as with tomato wilt caused by *Fusarium oxysporum* f. sp. *lycopersici*, *F. solani*, *Rhizoctonia solani*, and *Sclerotium rolfsii* [11]; cucumber damping-off caused by *Pythium aphanidermatum* [12]; wheat root rot caused by *Bipolaris sorokiniana* and *Fusarium* spp. [13]; and corn sheath blight caused by *Rhizoctonia* species [14]. Evidently, the soil microbial community is an important factor for plant health [15]; thus, the balance between beneficial and deleterious rhizosphere microorganisms is critical for healthy crops [16].

Wheat is the third most important cereal crop worldwide, following rice and corn. However, wheat crops are affected by serious diseases during all growth stages, with nearly 200 different types of wheat diseases having been reported around the worldwide. China, as a large agricultural country, accounts for 17% and 16% of the total world wheat production and consumption, respectively [17], with wheat annually grown on >24 million hectares; however, there are more than 70 diseases and insect pests affecting wheat, with approximately 6.4 million hectares of wheat annually affected [18]. According to estimates of the United Nations' Food and Agriculture Organization, wheat suffers drastic yield losses of 5 billion tons a year worldwide owing to diseases, with fungal diseases constituting the leading cause of disease. Soil-borne fungal diseases of wheat mainly include wheat common root rot, wheat take-all, root and stem base rot, and wheat sharp eyespot [19].

Wheat common root rot, mainly caused by *Bipolaris*, *Fusarium*, *Rhizoctonia*, and *Pythium* species, is a worldwide disease occurring at different extents in wheat-producing countries, which in severe cases may reduce the yield by 20–50% [20]. Of the causative organisms, *B. sorokiniana* is the dominant pathogen in China. Wheat sharp eyespot, primarily caused by *R. cerealis*, has become one of the most serious wheat diseases in China [21], posing a new threat to global wheat production [22]. Clarkson and Cook observed that in England and Wales, slight sharp eyespot infection had little effect on yield; moderate infection significantly reduced yield per ear and 1000-grain weight by 5% and 4%, respectively, and that severe infection significantly reduced yield per ear and its components, grain number per ear, and 1000-grain weight, by 26%, 20%, and 11%, respectively [23]. Wheat take-all, also known as black foot disease, is caused by *Gaeumannomyces graminis* var. *tritici* and is one of the most destructive wheat root diseases worldwide [24]. Following wheat take-all infection, the root system is destroyed, reducing grain yield by approximately 10–50% [25]. Wheat stem base rot, caused by *Fusarium* spp., is a wide-spread disease affecting wheat production [26]. *F. pseudograminearum*, *F. graminearum*, and *B. sorokiniana* are the dominant pathogens of stem base rot disease in major winter wheat production areas in China; these fungal species mainly damage the stem base and root crown of wheat and produce some necrotic spots at the stem base, resulting in a series of symptoms such as root rot, seedling withering, and white spike.

Over the past few decades, excessive amounts of chemical fertilizers and pesticides have been used to maintain a high level of agricultural productivity. However, this is not only toxic to humans but also harmful to the environment and results in an imbalance within the soil microbial community and a rapid increase in the spread of resistance genes [27]. This has encouraged researchers to focus their attention on biological control strategies, such as microbial inoculation, to either replace or reduce the use of agrochemicals.

Microorganism-based inoculants can act as biofertilizers and biocontrol agents for enhancing nutrient uptake, promoting crop growth, altering microbial community structure, and reducing soil-borne diseases. In recent years, biological control has achieved some success in different crops, and numerous studies have assessed the effect of biological amendments on the prevention and control of soil-borne diseases of crops.

For microbial inoculants, the ability to establish and maintain sufficient population size within the rhizosphere is a critical prerequisite for the control of soil-borne wheat diseases. In this regard, using indigenous microorganisms to develop biocontrol agents is substantially advantageous. In this study, the efficacy of microbial inoculants BIO-1 and BIO-2 in reducing soil-borne wheat diseases and their effect on soil microbial community structure were evaluated in a plot test. The aim of this study was to develop microbial inoculants for application in local arable land and to explore and improve our understanding of the biocontrol mechanisms of the microbial inoculants BIO-1 and BIO-2 from a rhizosphere microbiota ecology perspective.

## 2. Materials and Methods

### 2.1. Biocontrol Strain and Culture Medium

The microbial inoculants BIO-1 and BIO-2 were enriched with *Paenibacillus jamilae* HS-26 (3.4 × 10^9^ cfu g^−1^) and *Bacillus amyloliquefaciens* subsp. *plantarum* XH-9 (18.0 × 10^10^ cfu g^−1^), respectively. Both *P. jamilae* HS-26 and *B. amyloliquefaciens* subsp. *plantarum* XH-9 have efficient antagonistic activity and other growth-promoting characteristics, which have been published in our previous study [28].

### 2.2. Pathogen Inoculum Production

*F. graminearum* F0609, provided by the Jiangsu Academy of Agricultural Sciences, was used as the pathogen in this study. The pathogen inoculants were prepared by inoculating plate-cultured *F. graminearum* F0609 into sterilized wheat grain (high temperature moist heat sterilization, 121°C for 30 min) at a ratio of 1 : 1000 at 28 ± 2°C for 15–20 d.

### 2.3. Experimental Design

An experimental plot system was established in Nanjing Liuhe area of the Jiangsu Province, China, between October 2017 and March 2018.

This study used a completely randomized block design with three replicates per treatment (CK, T1, and T2), each replicate consisting of an area of 40 m^2^ (8 m length × 5 m width). Wheat seeds (Ningmai26) were surface-sterilized with 1% sodium hypochlorite for 5 min, washed 3–5 times with sterile water, and sown artificially on the plot at 0.6 kg per 40 m^2^ on October 20, 2017. The average temperature is 16°C, and the soil relative humidity is 38%. At the jointing stage, the seedlings were inoculated with pathogen inoculants (1.2 kg per 40 m^2^ soil). After 2 days, the wheat seedlings in the treatment groups were first irrigated with BIO-1 and BIO-2 (which were previously dissolved in water at 0.3 kg per 40 m^2^) on February 27, 2018, and then on March 13, 2018; the wheat seedlings in the control group were irrigated with the same volume of tap water. The basal fertilizer (45% Yangfeng compound fertilizer, N14-P16-K15; 1.2 kg per 40 m^2^) was applied prior to preplanting; the nitrogen fertilizer (urea, 1.5 kg per 40 m^2^) was applied during the green up period.

### 2.4. Effects of Microbial Inoculants on Disease

White heads in wheat, the main disease characteristic, were investigated at the end of grouting. Each treatment had three replicates, three sampling points were selected at random for each replicate, and 100 wheat seedlings were surveyed for each sampling point. The number of white heads was counted, and the relative biocontrol efficacy was calculated based on white head rate in the plot [29] using the following formulae.

(1) $$\begin{array}{c} \text{Disease incidence}=\frac{\text{The number of diseased plants}}{\text{Total number of wheat seedling}}\times 100\%,\\ \text{Relative efficacy}=\frac{\text{disease incidence in control}-\text{disease incidence in treatment}}{\text{disease incidence in control}}\times 100\%. \end{array}$$

### 2.5. Soil Sample Collection

Soil tightly adhered to the roots was regarded as rhizosphere soil, which was collected by gentle shaking. At the end of grouting, the wheat seedlings were uprooted, and the rhizosphere soil samples were collected from five random soil cores from each plot; each soil core had 3–5 wheat seedlings. Every five samples were pooled to yield one composite sample per replicate, thoroughly homogenized, and passed through a 2 mm sieve. The processed soil samples were stored at -80°C.

### 2.6. Genomic DNA Preparation and Illumina MiSeq Sequencing

Total soil genomic DNA was extracted from 0.5 g of soil using the E.Z.N.A. Soil DNA Kit (Omega Bio-tek, Norcross, GA, USA). Purified DNA was stored at -80°C prior to PCR amplification. The bacterial 16S rRNA and fungal rDNA-ITS genes were amplified from the total soil genomic DNA using primers 515F/907R (16S rDNA V3-V4 genes) and ITS1F/2043R (ITS region gene), respectively. Both the forward and reverse primers were tagged with an adapter, an eight-base sequence unique to each sample. The 20 *μ*L reaction mixture consisted of 4 *μ*L of 5 × FastPfu Buffer, 2 *μ*L of 2.5 mM dNTPs, 0.8 *μ*L of each primer (5 *μ*M), 0.4 *μ*L of FastPfu Polymerase, and 10 ng of template DNA. The thermal cycling PCR parameters included an initial denaturation step at 95°C for 2 min; followed by 25 cycles at 95°C for 30 s, 55°C for 30 s, and 72°C for 30 s; and a final extension at 72°C for 5 min. Amplicons were extracted, purified, pooled in equimolar concentrations, and then paired-end sequenced (2 × 250) on an Illumina MiSeq platform (Illumina, USA) by Majorbio Bio-pharm Technology Co., Ltd. (Shanghai, China) according to standard protocols.

### 2.7. Sequence Processing

For processing of the sequencing data, raw FASTQ files were demultiplexed and quality-filtered using QIIME (version 1.9.1, http://qiime.sourceforge.net/); chimeric sequences were identified and removed using UCHIME (version 4.2.40, http://drive5.com/usearch/manual/uchime algo.html) [30]; the remaining high-quality sequences were clustered with a 97% similarity level cut-off using UPARSE (version 7.1; http://drive5.com/uparse/) to generate operational taxonomic units (OTUs); Venn diagrams were generated using the Venn diagram program [31]; alpha diversity indices were calculated using the Mothur program with an OTU cut-off of 0.03 [32]; the 16S rRNA reads were assigned to bacterial taxonomic groups using the Ribosomal Database Project (RDP) classifier (http://rdp.cme.msu.edu/) against the Silva (SSU123) 16S rRNA database, with a confidence threshold of 70%; the taxonomy of each rDNA-ITS gene sequence was analyzed using the RDP Classifier against the UNITE 7.0/ITS database with a confidence threshold of 70% [33]; principal coordinate analysis (PCoA) was based on the OTU Bray–Curtis dissimilarity matrices at a 97% cut-off, and the hierarchical cluster tree was constructed based on a distance matrix calculated using the unweighted UniFrac algorithm [34].

### 2.8. Statistical Analysis

All experiments were performed in triplicate, and all statistical analyses were performed using the SAS version 8.0 software (SAS Institute, Inc.). Differences in mean values were considered significant when *P* was <0.05.

## 3. Results

### 3.1. Effects of Microbial Inoculants on Disease

Wheat disease incidence was measured at the end of grouting. For each replicate, 300 wheat seedlings, diseased plants, disease incidence, and relative efficacy, were surveyed (Table 1). The number of diseased plants was significantly lower in the treatment groups than in the control group (*P* < 0.05). The relative efficacies of BIO-1 and BIO-2 were up to 82.5% and 83.9%, respectively. Based on these results, we collected rhizosphere soil samples from the treatment and control groups to explore the underlying biocontrol mechanisms of microbial inoculants at rhizosphere microbial community composition level.

### 3.2. Processing of Illumina MiSeq Sequencing Data

**Figure 1:**
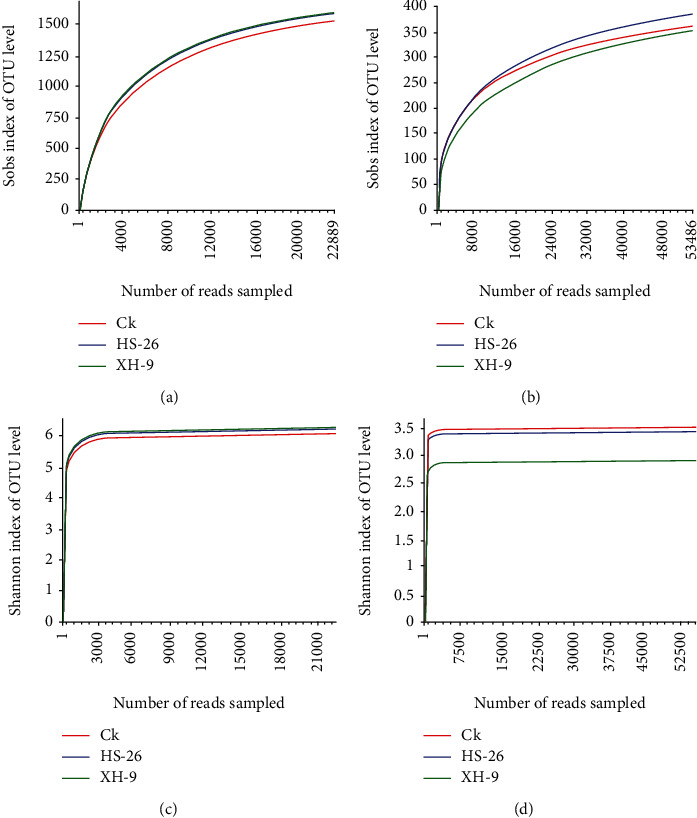
Bacterial and fungal rarefaction curves and Shannon curves depicting the effect of microbial inoculant treatment (T1 and T2) and control groups on the number of OTUs: (a) rarefaction curves of bacteria from the T1 and T2 treatment and control groups; (b) rarefaction curves of fungi from the T1 and T2 treatment and control groups; (c) Shannon curves of bacteria from the T1 and T2 treatment and control groups; (d) Shannon curves of fungi from the T1 and T2 treatment and control groups.

A total of 402,798 valid reads from the 16S rRNA gene V3 and V4 variable regions and 518,973 valid reads from the ITS region were obtained from the nine soil samples, with average lengths of 427 and 262 bp, respectively. More than 40,000 high-quality bacterial and 50,000 fungal sequences were obtained for each replicate for further analysis. These sequences were grouped into 5,558 bacterial OTUs and 1,451 fungal OTUs using a 97% nucleotide sequence identity threshold. All rarefaction curves (Figure 1) approached the saturation plateau with an increase in sequencing number, indicating that the sequencing capability and sequenced reads were sufficiently extensive to capture the complete diversity of these communities. The *α*-diversity values of the soil microbial communities are detailed in Table 2. The bacterial community richness indices (ACE and Chao) and diversity indices (Shannon) were lower in the control group than in the T1 and T2 treatment groups, indicating a higher richness and diversity of bacterial communities in the treatment groups. The Simpson index showed a similar trend for bacterial community structure. Although there were no significant differences in fungal community richness indices following treatment with BIO-1 and BIO-2, higher ACE and Chao values were observed in the T1 and T2 groups than in the control group. Additionally, fungal diversity significantly decreased following the application of BIO-2, similar to the Shannon index results.

### 3.3. Microbial Community Composition

**Figure 2:**
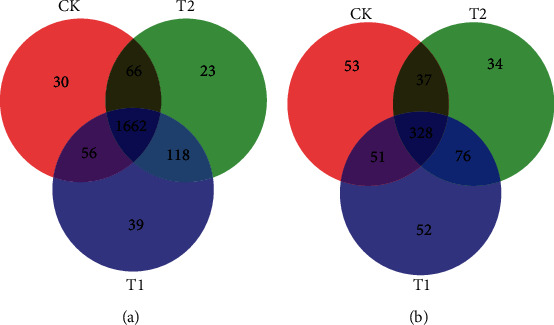
Venn diagrams showing the unique and shared OTUs (3% distance level) between the microbial inoculant treatment and control groups: (a) a Venn diagram of bacteria from the T1 and T2 treatment and control groups; (b) a Venn diagram of fungi from the T1 and T2 treatment and control groups.

To compare the relationships between the control and the treatments, Venn diagrams were constructed based on OTU levels (Figure 2). A total of 5,558 bacterial OTUs and 1,451 fungal OTUs were obtained from the treatment and control groups combined. Bacterial OTU analysis indicated that 1,662 OTUs were common to all samples; 1,875, 1,869, and 1,814 OTUs belonged to the T1, T2, and control groups, respectively. Fungal OTU analysis indicated that 328 OTUs were common to all samples; 507, 475, and 469 fungal OTUs belonged to the T1, T2, and control groups, respectively. Of these, 39 bacterial OTUs and 52 fungal OTUs were unique to the T1 group; 23 bacterial OTUs and 34 fungal OTUs were unique to the T2 group, and 30 bacterial OTUs and 53 fungal OTUs were unique to the control group. To further investigate the composition of the bacterial and fungal communities, all bacterial and fungal sequences were classified at the phylum level down to the genus level. The numbers of bacterial and fungal phyla, classes, orders, families, genera, and species are detailed in Table 3.

### 3.4. Soil Bacterial Community Composition

**Figure 3:**
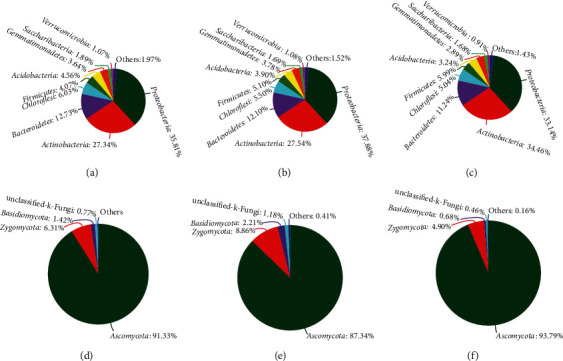
Relative abundance (%) of all bacteria and fungi community composition at the phylum level in the microbial inoculant treatment and control groups: (a–c) relative abundance of all detected bacterial phyla in the control and T1 and T2 treatment groups, respectively; (d–f) the relative abundance of all detected fungal phyla in the control and T1 and T2 treatment groups, respectively.

**Figure 4:**
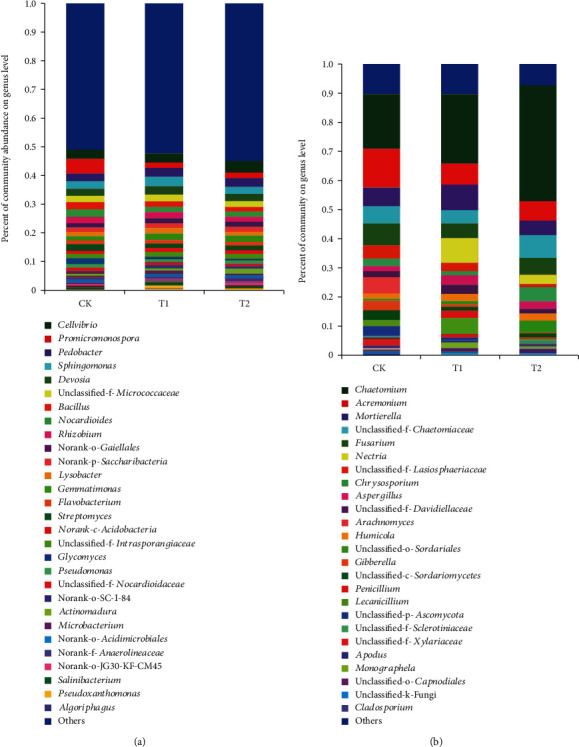
Relative abundance (%) of all bacteria (a) and fungi (b) at the genus level in the rhizosphere soil of microbial inoculant treatment (T1 and T2) and control groups.

A total of nine bacterial phyla were detected, with Proteobacteria, Actinobacteria, and Bacteroidetes representing the most dominant phyla, followed by Chloroflexi, Firmicutes, Acidobacteria, Gemmatimonadetes, Saccharibacteria, and Verrucomicrobia (Figures 3(a)–3(c)). The abundance of Proteobacteria and Actinobacteria in the T1, T2, and control groups was 37.88%, 33.14%, and 35.81% and 27.54%, 34.46%, and 27.34%, respectively. The phylum Bacteroidetes abundance was 12.10%, 11.24%, and 12.73% in the T1, T2, and control groups, respectively. At the genus level, it was found that although bacterial composition was similar in the T1, T2, and control groups, the distribution of each genus differed by soil sample (Figure 4(a)). *Sphingomonas*, *Bacillus*, *Nocardioides*, *Rhizobium*, *Streptomyces*, *Pseudomonas*, and *Microbacterium* were more abundant in the T1 and T2 groups than in the control group. Furthermore, the abundance of *Devosia*, *Lysobacter*, and *Gemmatimonas* was higher in the T1 group than in the control group and that of *Glycomyces* and *Promicromonospor*a was higher in the T2 group than in the control group (Table 4).

### 3.5. Soil Fungal Community Composition

Ascomycota was the most abundant fungal phylum across all samples, followed by Zygomycota and Basidiomycota (Figures 3(d)–3(f)). The abundance of Ascomycota was 87.34%, 93.79%, and 91.33% in the T1, T2, and control groups, respectively. At the genus level (Figure 4(b)), *Arachnomyces* and *Cladosporium* were only identified in the control group, with an abundance of 5.44% and 1.03%, respectively, while *Nectria* was only observed in the T1 and T2 groups, with an abundance of 8.16% in T1 and 3.01% in T2. Additionally, *Chaetomium* clearly increased in the T1 and T2 groups (23.98% and 39.70%, respectively) compared with that in the control (18.91%); *Aspergillus* and *Humicola* also demonstrated a slight increase, while the abundance of *Acremonium* decreased in the T1 and T2 groups (7.27% and 6.05%, respectively) compared with that in the control group (13.28%). Interestingly, the abundance of both *Fusarium* and *Gibberella* in soil samples decreased following microbial inoculant treatment, accounting for 5.11% and 0.84% in T1, 5.75% and 0.50% in T2, and 7.60% and 3.60% in the control groups, respectively. Conversely, the abundance of *Penicillium* was much higher in the T1 (3.02%) and T2 (0.86%) groups than in the control group (0.65%) (Table 4).

### 3.6. Comparison of Microbial Community between the Different Treatment Groups

**Figure 5:**
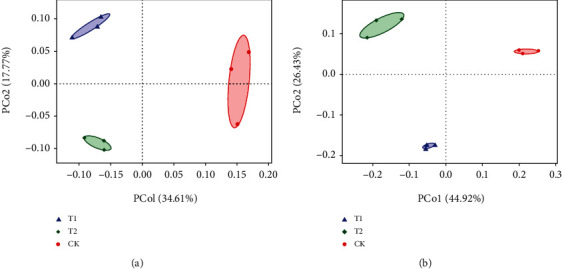
Principal coordinate analysis (PCoA) based on the OTU Bray–Curtis dissimilarity matrices at a 97% cut-off was used to investigate the differences or similarities in rhizosphere microbial community between the microbial inoculant treatments and control groups: (a) PCoA of the bacterial communities; (b) PCoA of the fungal communities.

PCoA was performed to analyze the differences or similarities (Bray–Curtis index) in rhizosphere microbial community between microbial inoculant-treated groups and control groups. Both the bacterial and fungal community structures of the control group were clearly separated from those of the treatment groups (T1 and T2) along the PC1 axis (34.61%) for bacteria and PC1 axis (44.92%) for fungi (Figure 5). The control groups resulted in higher PC1 values than the two treatment groups. On the PC2 axis, the T1 groups showed higher PC2 values for bacteria and lower PC2 values for fungi than the control group, while the T2 groups showed lower PC2 values for bacteria and higher PC2 values for fungi. Hence, the T1 groups were obviously separated from the T2 groups along the PC2 axis (17.77%) for bacteria and PC2 axis (26.43%) for fungi. These results indicate that the first component was differentiated based on microbial inoculant treatment or no treatment, while the second component was differentiated based on the species of microbial inoculant (*P. jamilae* or *B. amyloliquefaciens* subsp. *plantarum*). Furthermore, permutational multivariate analysis of variance of the microbial communities between the microbial inoculant-treated groups and control groups was in agreement with the PCoAs; microbial inoculants BIO-1 and BIO-2 had a significant effect on rhizospheric microbial communities when using a Bray–Curtis distance metric (69.98%, *P* < 0.05).

**Figure 6:**
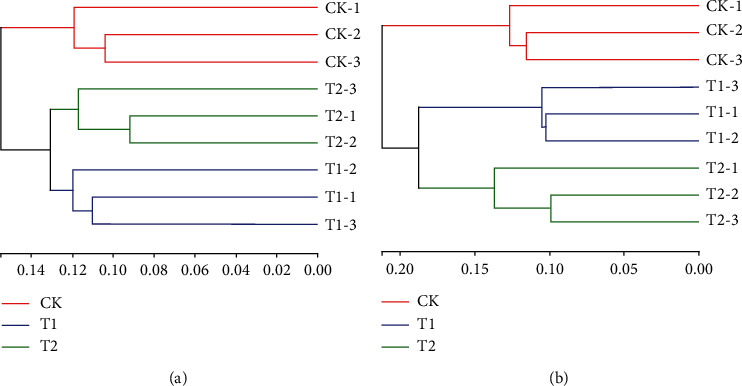
Hierarchical cluster tree constructed based on the distance matrix calculated using the unweighted UniFrac algorithm for the microbial inoculant treatment (T1 and T2) and control groups: (a) hierarchical cluster tree of the bacterial communities; (b) hierarchical cluster tree of the fungal communities.

Moreover, all soil samples from the treatment and control groups were compared, and a hierarchical cluster tree was constructed (Figure 6). This tree also showed obvious differences between the microbial inoculant treatments and control groups. Both the bacterial and fungal community structures of the T1 and T2 groups clustered together but were separated from those of the control groups. Taken together, these results indicate that the microbial community structure was clearly altered following microbial inoculant treatment.

## 4. Discussion

Biocontrol by inoculation with beneficial microbes is a proven strategy for reducing the negative effects of soil-borne pathogens [35–37]. Qiao et al. found that PGPR strain *B. subtilis* PTS-394 supported the growth of tomato plants and suppressed soil-borne diseases [38]. Moussa et al. reported that using fluorescent *Pseudomonas* MAA10 and *B. subtilis* MAA03 separately or in a mixture as a biocontrol agent efficiently suppressed wheat root-invading pathogens and significantly affected the growth parameters of wheat cultivars [39]. Bo et al. demonstrated that the biocontrol agent *B. amyloliquefaciens* ZM9 was effective in the control of tobacco bacterial wilt, with increased relative abundance of PGPR in the biocontrol groups [40]. Wu et al. found that the application of microbial fertilizer altered bacterial abundance and community structure; in particular, it increased the abundance of indigenous microbial groups with notable antifungal activity [41].

However, the efficiency of biocontrol is sensitive to a variety of biological and nonbiological factors. Hence, elucidating biocontrol mechanisms under actual conditions could facilitate the evaluation and improvement of soil-borne pathogen biocontrol in agriculture. In the current study, we performed a plot experiment to evaluate the effect of microbial inoculants BIO-1 and BIO-2 in reducing soil-borne wheat diseases and on the rhizosphere soil microbial community composition of wheat crops. Importantly, the wheat disease incidence rate clearly decreased in both treatment groups compared with that in the control group, indicating that BIO-1 and BIO-2 can effectively reduce the occurrence of disease. MiSeq sequencing data analysis demonstrated that both BIO-1 and BIO-2 significantly increased bacterial diversity and richness in the rhizosphere (Table 2). Zhang et al. reported that ectomycorrhizal (ECM) fungal inoculation significantly increased the ectomycorrhizal colonization and influenced bacterial functional diversity compared with that in noninoculated Chinese pine seedlings [42]. Chen et al. reported that the inoculation of *Azotobacter* can change the soil enzyme activities, regulate the soil bacterial functional community, and increase the total bacterial metabolic activity [43].

The relative abundance of some beneficial genera, including *Sphingomonas*, *Bacillus*, *Nocardioides*, *Rhizobium*, *Streptomyces*, *Pseudomonas*, and *Microbacterium*, increased in soil treated with microbial inoculants. Of these, members of *Streptomyces* have been studied and applied as producers of diverse and important metabolites including antibiotics, herbicides, and enzymes [44, 45]. *Bacillus* spp. are also considered as beneficial bacteria based on their ability to promote plant growth and inhibit the growth of phytopathogens [46, 47]. *Rhizobium* and *Devosia* spp. are known to be involved in nitrogen fixation [48]. *Lysobacter* and *Glycomyces* have been shown to possess antagonistic activities against many soil-borne diseases by producing a series of metabolites and extracellular enzymes [49, 50]. Wang et al. demonstrated that *Bacillus fusiformis* L13 used as compound microbial fertilizer increased the proportion of Proteobacteria and *Firmicutes* and increased the amount of beneficial bacteria including Actinomycetales and Bacillales [51].

At the fungal genus level, we found that *Chaetomium*, *Nectria*, *Aspergillus*, *Humicola*, and *Penicillium* were more abundant in the T1 and T2 groups than in the control group. Of these genera, *Chaetomium* and *Penicillium* have been used as biocontrol agents because of their numerous secondary metabolites [52, 53]. *Humicola* and *Aspergillus* spp. are also considered to be beneficial soil fungi, as some representatives have been used as important enzyme producers in the renewable energy industry [54, 55]. Additionally, *Aspergillus* spp. have been widely used as phosphate-solubilizing fungi based on their ability to synthesize organic acids such as oxalic, tartaric, and citric acid [56]. As predicted, we found that the abundance of the pathogenic fungi *Fusarium* and *Gibberella* was lower following treatment with microbial inoculants. Of note, all *Gibberella* species are sexual states of *Fusarium* species, and many *Gibberella* species are destructive plant pathogens [57]. *Gibberella zeae* is one of the most economically harmful cereal pathogens; this species causes head blight of wheat (*Triticum aestivum* L.), barley, and other small grains and also infect maize ears and stalks and a variety of other plants worldwide [58]. *Fusarium* includes a large number of species affecting agriculture and plant protection, constituting an enormous impact on plant and food production [59], such as wheat head blight caused by *F. graminearum* [60], banana *Fusarium* wilt disease caused by *F. oxysporum* f. sp. *cubense* [61], and bakanae disease of rice caused by *F. moniliforme* Sheld. [62].

Both PCoA and the hierarchical cluster tree (Figures 5 and 6) showed that the microbial inoculant treatment and control groups were separate from each other. A possible explanation for this phenomenon is that beneficial microorganisms are generally saprophytic and rely on soil nutrients, while host-specific pathogens are parasitic microorganisms and rely on host plants [63]. In this study, BIO-1 and BIO-2 enriched with the antagonistic bacteria *P. jamilae* HS-26 and *B. amyloliquefaciens* subsp. *plantarum* XH-9 were introduced into the wheat rhizosphere in the T1 and T2 groups. Both HS-26 and XH-9 strains also possess a series of growth-promoting characteristics, such as nitrogen fixation, phytohormone production, and phosphate and potassium-solubilization, which contributed to increased nutrient availability, and thus, the beneficial genera in the microbial inoculant treatment groups were stimulated. In turn, the healthy plants released large amounts of exudates into the rhizosphere, stimulating the metabolic activities of the microbes in the rhizosphere, likely resulting in a change in the composition of the indigenous rhizosphere microbial communities [64]. Furthermore, both *P. jamilae* HS-26 and *B. amyloliquefaciens* subsp. *plantarum* XH-9 can directly suppress pathogen propagation by secreting antibiotic metabolites and competing for the same microbial niche in the rhizosphere; hence, the species and relative abundance of pathogens differed between the treatment and control groups. Additionally, we observed differences in the bacterial and fungal communities between the T1 and T2 groups, especially in the effect on fungal community structure. This difference may be owing to *P. jamilae* HS-26 and *B. amyloliquefaciens* subsp*. plantarum* XH-9, which belong to different genera and hence possess different PGP characteristics, secondary metabolites, colonization patterns, and synergy and competition modes with indigenous microorganisms, sensitivity to environmental factors, and other unexplored properties.

## 5. Conclusion

In summary, these results indicate that application of BIO-1 and BIO-2 reduced disease incidence and affected the composition and structure of indigenous microbial communities. Bacterial diversity and richness were significantly higher in both the T1 and T2 groups than in the control group, and fungal diversity significantly decreased following the application of BIO-2. The relative abundance of beneficial microbes, such as *Rhizobium*, *Streptomyces*, *Pseudomonas*, *Chaetomium*, and *Penicillium*, increased in the T1 and T2 groups, while that of soil-borne plant pathogenic fungi, including *Fusarium* and *Gibberella*, decreased in the T1 and T2 groups. These results indicate that the well-known plant-associated bacteria accumulated more easily in the wheat rhizosphere of the microbial inoculant treatment groups and consequently reduced disease severity. This observation could be attributed to the suppression of pathogen proliferation by the microbial inoculants by producing secondary metabolites that promote the accumulation of well-known plant-associated bacteria. However, further studies have to be carried out to assess and validate the role of antibiotic and hydrolytic activities produced by microbial inoculants in promoting the growth of beneficial microbes in soil rhizosphere. Overall, our results showed that BIO-1 and BIO-2 are suitable for local application in arable land and have potential for future promotion.

## Figures and Tables

**Table 1:** Disease prevention efficacy by microbial inoculants.

Index	CK	T1	T2
Total number of wheat seedlings	300	300	300
Diseased plants	149 ± 1 5.5a	26 ± 5.7b	24 ± 3.3b
Disease incidence	49.7%	8.7%	8.0%
Relative efficacy	—	82.5%	83.9%

Values are presented as means ± SD (*n* = 3). Means sharing a common letter within the same row are not significantly different at *P* < 0.05. “T1” denotes wheat seedlings treated with *F. graminearum* F0609 and BIO-1; “T2” denotes wheat seedlings treated with *F. graminearum* F0609 and BIO-2; “CK” denotes wheat seedlings treated with *F. graminearum* F0609 and an equal volume of sterile water.

**Table 2:** Diversity and richness indices of bacterial and fungal communities from the microbial inoculant treatment and control groups.

Index	Bacteria			Fungi		
	CK	T1	T2	CK	T1	T2
Sobs	1526.00 ± 40.93a	1597.67 ± 13.58a	1583.00 ± 46.36a	359.67 ± 9.87a	382.67 ± 24.54a	351.33 ± 22.37a
Shannon	6.05 ± 0.09b	6.20 ± 0.12ab	6.27 ± 0.07a	3.50 ± 0.06a	3.42 ± 0.09a	2.90 ± 0.10b
Simpson	0.007 ± 0.00a	0.005 ± 0.00b	0.004 ± 0.00b	0.07 ± 0.004b	0.08 ± 0.007b	0.17 ± 0.04a
ACE	1725.73 ± 42.83b	1834.19 ± 45.27a	1735.87 ± 51.38b	410.29 ± 18.45a	440.20 ± 22.01a	422.74 ± 35.51a
Chao	1737.05 ± 55.08b	1856.92 ± 47.00a	1769.81 ± 48.42ab	405.78 ± 17.86a	435.44 ± 23.88a	412.90 ± 2 1.53a
Coverage	0.9853	0.9867	0.9873	0.9988	0.9986	0.9985

The Chao and ACE values are indicators of species richness. The Shannon and Simpson values are indicators of species diversity. Values are presented as means ± SD (*n* = 3). Means sharing a common letter within the same column are not significantly different at *P* < 0.05.

**Table 3:** Number of bacterial and fungal sequences from the microbial inoculant treatment and control groups classified at the phylum level down to the genus level.

Levels	Bacteria			Fungi		
	CK	T1	T2	CK	T1	T2
Phylum	26	29	28	6	6	6
Class	57	63	61	16	17	16
Order	133	139	138	47	50	47
Family	267	278	272	97	99	99
Genus	550	559	553	170	176	170
Species	994	1,024	1,017	268	270	262
OTUs	1,823	1,876	1,868	469	507	475

**Table 4:** Relative abundance of all bacteria and fungi at the genus level in the microbial inoculant treatment and control groups.

Genus	Bacteria			Genus	Fungi		
	CK	T1	T2		CK	T1	T2
*Cellvibrio*	3.77%	3.08%	3.15%	*Chaetomium*	18.91%	23.98%	39.70%
*Promicromonospora*	2.37%	2.11%	4.80%	*Acremonium*	13.28%	7.27%	6.05%
*Pedobacter*	3.10%	2.81%	2.89%	*Mortierella*	6.29%	8.82%	4.75%
*Sphingomonas*	2.37%	3.38%	2.71%	*Fusarium*	7.60%	5.11%	5.75%
*Devosia*	2.57%	2.83%	2.25%	*Nectria*	0	8.16%	3.01%
*Bacillus*	1.99%	2.08%	2.39%	*Chrysosporium*	2.28%	1.02%	4.82%
*Nocardioides*	1.57%	1.82%	2.43%	*Aspergillus*	1.80%	3.26%	2.18%
*Rhizobium*	1.77%	2.03%	1.90%	*Arachnomyces*	5.44%	0	0
*Lysobacter*	1.59%	2.13%	1.30%	*Humicola*	1.78%	2.64%	1.89%
*Gemmatimonas*	1.71%	1.84%	1.37%	*Gibberella*	3.60%	0.84%	0.50%
*Flavobacterium*	1.82%	1.69%	1.27%	*Penicillium*	0.65%	3.02%	0.86%
*Streptomyces*	1.12%	1.23%	2.44%	*Lecanicillium*	1.73%	2.34%	0.45%
*Glycomyces*	0.96%	0.81%	1.99%	*Apodus*	0.90%	1.99%	0.76%
*Pseudomonas*	1.03%	1.14%	1.17%	*Monographella*	0.61%	1.94%	0.95%
*Actinomadura*	1.53%	0.65%	0.89%	*Cladosporium*	1.03%	0	0
*Microbacterium*	0.87%	1.04%	1.13%				
*Salinbacterium*	1.02%	1.22%	0.58%				
*Pseudoxanthomonas*	0.57%	1.17%	0.48%				
*Algoriphagus*	0.46%	1.30%	0.42%				
*Haloactinopolyspora*	1.00%	0.46%	0.62%				

## Data Availability

All data generated or analyzed during this study are included in this published article.

## Abbreviations

ACCD: Aminocyclopropane-1-carboxylate deaminase
PGPR: Plant growth-promoting rhizobacteria
PCoA: Principal coordinate analysis
RDP: Ribosomal database project
IAA: Indole acetic acid.
